# Rapid single-flux-quantum and adiabatic quantum-flux-parametron cell libraries using a 1 kA/cm^2^ niobium fabrication process

**DOI:** 10.1038/s41598-025-20666-7

**Published:** 2025-11-21

**Authors:** Taiki Yamae, Yuki Hironaka, Shuichi Nagasawa, Yuki Yamanashi, Nobuyuki Yoshikawa, Naoki Takeuchi

**Affiliations:** 1 Global Research and Development Center for Business by Quantum-AI Technology, National Institute of Advanced Industrial Science and Technology (AIST), Tsukuba, 305-8568 Ibaraki Japan; 2 Institute of Advanced Sciences, Yokohama National University, Yokohama, 240-8501 Kanagawa Japan; 3 Department of Electrical and Computer Engineering, Yokohama National University, Yokohama, 240-8501 Kanagawa Japan; 4 Graduate School of System Informatics, Kobe University, Kobe, 657-8501 Hyogo Japan

**Keywords:** Electrical and electronic engineering, Superconducting devices

## Abstract

Superconductor logic families can operate with small power dissipation and are thus suitable as building blocks for various computing systems. In some applications, superconductor logic circuits should be designed using Josephson junctions with low *I*_c_ values (*I*_c_: critical current). For instance, lowering *I*_c_ values enables qubit interface circuits to operate with very small power dissipation at ~10 mK and stochastic electronics to easily induce stochastic operations. In this study, we develop the AIST 1 kA cm^−2^ Nb planarized process (1KP) with a minimum critical current of 10 µA, dedicated to the design of qubit interface circuits and stochastic electronics. We also develop rapid single-flux-quantum (RSFQ) and adiabatic quantum-flux-parametron (AQFP) cell libraries using the 1KP. The power dissipation of RSFQ logic using the 1KP can be reduced to 3.2% of that for conventional RSFQ logic by reducing both *I*_c_ values and a bias voltage. Furthermore, the amount of supply currents for AQFP circuits using the 1KP can be reduced to ~40% of that for conventional AQFP circuits due to a large mutual inductance between AQFP gates and excitation lines, which results from a reduction in *I*_c_ and an increase in inductances. We demonstrate RSFQ and AQFP circuits fabricated by the 1KP at 4.2 K. These results indicate that RSFQ and AQFP circuits using the 1KP have the potential to be used for the design of qubit interface circuits and stochastic electronics.

Superconductor logic families^1–6^ can operate with small power dissipation and have thus been studied to develop various energy-efficient systems. To suppress errors caused by thermal noise at a typical operating temperature of 4.2 K, the critical currents of Josephson junctions (JJs) in superconductor logic families are set to 100–300 µA^7,8^ using Nb fabrication processes^9,10^ with a critical current density (*J*_c_) of ~10 kA/cm^2^. This ensures that the Josephson energy (*I*_c_Φ_0_/2π = 3.29 × 10^−20^J for *I*_c_ = 100 µA) is much larger than thermal energy (*k*_B_*T* = 5.80 × 10^−23^J for *T* = 4.2 K), where *I*_c_ is the critical current of a JJ, Φ_0_ is a flux quantum, *k*_B_ is the Boltzmann constant, and *T* is temperature. Low-error-rate, high-speed superconductor digital circuits have been demonstrated at 4.2 K^3,11,12^ using various logic families, such as rapid single-flux-quantum (RSFQ) logic^1^, reciprocal quantum logic^3^, and adiabatic quantum-flux-parametron (AQFP)^5,13^ logic.

In some applications using superconductor logic, smaller critical current values are preferable. Qubit interface circuits^14–18^ need to operate with extremely small power dissipation at sub-Kelvin temperature because of the poor cooling power of a dilution refrigerator, which is only ~10 µW at ~10 mK^19^. Thus, the critical current values of JJs in qubit interface circuits should be reduced to lower power dissipation, as far as *I*_c_Φ_0_/2π ≳ 1,000*k*_B_*T* at 10 mK (i.e., *I*_c_ ≳ 0.4 µA). Stochastic electronics^20–24^ perform stochastic operations, such as random number generation, by utilizing thermal fluctuations and typically use error-tolerant data formats^25^. Thus, the critical current values of JJs in stochastic electronics should also be reduced to easily induce stochastic operations and lower power dissipation (with errors accepted to some extent), as far as *I*_c_Φ_0_/2π ≳ *k*_B_*T* at 4.2 K (i.e., *I*_c_ ≳ 0.2 µA). The above discussion suggests that both qubit interface circuits and stochastic electronics should be designed using a low-*J*_c_ fabrication process with a minimum critical current of ~1 µA. In practice, too small critical current values are not preferable because very large inductors are required to design digital circuits; for instance, a 1-µA JJ requires a huge inductance *L* of ~2 nH to keep *LI*_c_ ~ Φ_0_. Therefore, we assume that the appropriate minimum *I*_c_ value is ~10 µA for the above two applications.

In this study, we develop a low-*J*_c_ fabrication process dedicated to the design of qubit interface circuits and stochastic electronics, which we call the AIST 1 kA cm^−2^ Nb planarized process (1KP). We selected a *J*_c_ value of 1 kA cm^−2^ to achieve the minimum *I*_c_ value of 10 µA for the minimum junction size of 1 µm^2^ in our fabrication facility. Other research groups also reported low-*J*_c_ (~1 kA cm^−2^ or even smaller) fabrication processes for qubit interface circuits^15,17,26–30^. In contrast, the 1KP is fully compatible with our standard RSFQ and AQFP designs^7,31–34^. Furthermore, we actually design RSFQ and AQFP cell libraries for the 1KP and demonstrate various logic cells fabricated by the 1KP. In the following sections, we first explain the device structure of the 1KP and measurement results for the characteristics of JJs fabricated by the 1KP. We then explain RSFQ and AQFP cell libraries using the 1KP, also revealing the advantages of using the 1KP (i.e., power dissipation and the amount of supply currents can be balanced for cryocooler applications). Finally, we present measurement results for RSFQ and AQFP circuits fabricated by the 1KP.

## Results and discussion

### Device structure and specifications of the 1KP

The AIST 10 kA cm^−2^ Nb planarized high-speed standard process (PHSTP)^34^ was developed for RSFQ and AQFP logic families by using the caldera planarization technique, which was developed for a nine-layer Nb process called the ADP2^9^. After that, several modified versions of the PHSTP have been developed depending on target applications. One is the 250P with a low *J*_c_ of 250 A/cm^2^, which was developed to design qubit interface circuits using RSFQ logic^29^. Another is the 1KP for qubit interface circuits and stochastic electronics using RSFQ and AQFP logic.

**Fig. 1:**
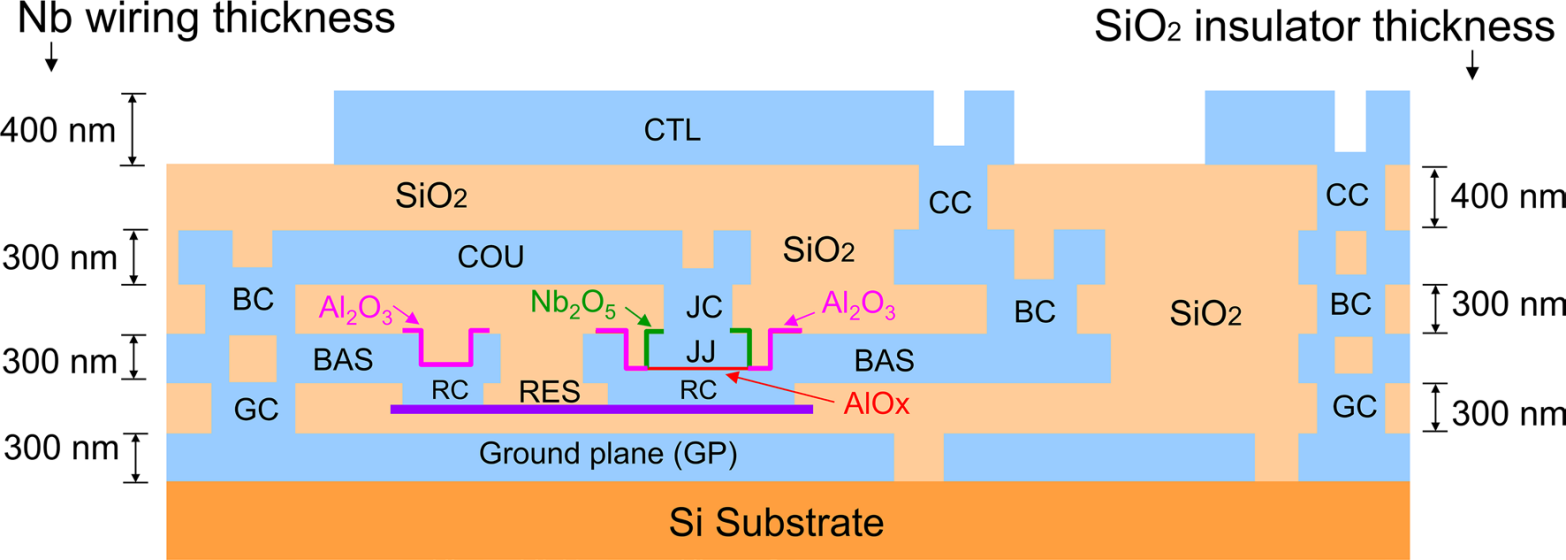
Device structure of the 1KP. JJ: Nb/AlO_x_/Nb junction; GP: Nb ground plane; BAS, COU, and CTL: Nb wiring layers; RES: Ti/Pd/Ti resistor (thickness: 2 nm/50 nm/4 nm); GC, RC, BC, JC, and CC: contacts between Nb layers. SiO_2_ thickness between GP and RES: 150 nm; that between RES and BAS: 150 nm; JJ upper layer thickness: 150 nm; Nb_2_O_5_: anodized Nb; Al_2_O_3_: anodized Al; AlO_x_: thermally oxidized Al.

Figure 1 shows a device structure of the 1KP, which is almost the same as that of the PHSTP. Table 1 shows comparison of PHSTP, 250P, and 1KP specifications. In the 250P and 1KP, the resistor material was changed from the Mo film to the Ti/Pd/Ti tri-layer film to obtain resistivity at 10 mK, which was originally introduced to the development of quantum annealing devices^35^. Thin Ti films are inserted in both sides of the Pd film to improve adhesion between Pd and SiO_2_. The target 1.2 Ω sheet resistance is obtained for a Pd thickness of approximately 50 nm. The *J*_c_ of Nb/AlO_x_/Nb junctions depends on the thickness of AlO_x_, which is controlled by oxidization pressure and time. The *J*_c_ of 1 kA/cm^2^ is obtained by a pressure of 15–17 Torr for Ar and 1%-O_2_ mixture gas and an oxidization time of 120 min.

### Characteristics of Josephson junctions fabricated by the 1KP

**Fig. 2:**
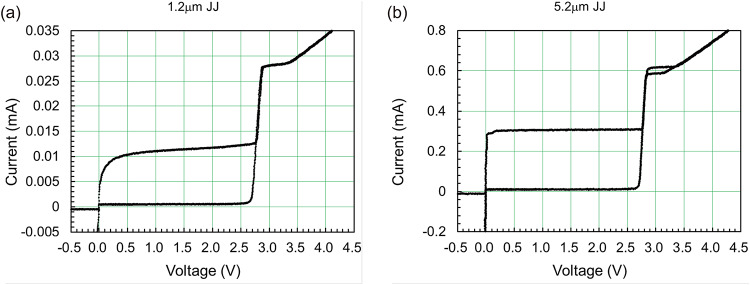
*I*-*V* characteristics of (**a**) a 1.2-μm-JJ array and (**b**) a 5.2-μm-JJ array, each comprising 1000 serially connected junctions (lot number: 1KP001 No. 1).

To evaluate junction characteristics, multiple test elementary groups (TEGs), each with 1000 serially connected JJs, were fabricated by the 1KP. Table 2 shows a summary of the junction characteristics for the wafer with a lot number of 1KP001 No. 1, where JJs with seven different sizes were measured at 4.2 K. By the extrapolation for the measured *I*_c_ values vs. junction sizes, *J*_c_ and shrinkage values were calculated to be 1248 A/cm^2^ and 0.244 μm, respectively, which are ~20% larger than the target values shown in Table 1. The junction area was calculated with the shrinkage value of 0.244 μm taken into account. The standard deviations of *I*_c_ scattering for small junctions (1.2, 1.6, and 2.2-µm JJs) are relatively large, compared to those for the PHSTP^34^. Figure 2(a) shows the current-voltage (*I*-*V*) characteristics of a JJ array comprising 1000 serially connected 1.2-μm JJs, which indicates that thermal noise at 4.2 K strongly suppressed the critical currents of small JJs and resulted in large *I*_c_ scattering. Therefore, the *I*_c_ scattering for small JJs will be improved by lowering operating temperature to 10 mK. On the other hand, the influence of thermal noise at 4.2 K on large junctions (4.2, 5.2, and 6.2-µm JJs) was relatively small, as indicated by the *I*-*V* characteristics of a JJ array comprising 1000 serially connected 5.2-μm JJs shown in Fig. 2(b). Excellent junction characteristics were observed for large junctions; the standard deviation for *I*_c_ scattering was less than 1%, *I*_c_*R*_n_ was more than 1.6 mV, and *I*_c_*R*_sg_ was more than 60 mV, where *R*_n_ is the resistance at 4.0 V due to normal currents and *R*_sg_ is the resistance of the sub-gap region due to quasi-particle currents.

**Table 1:** Comparison of PHSTP, 250P, and 1KP specifications.

	PHSTP	Modified version of PHSTP	
		250P	1KP
Critical current density of JJ	10 kA/cm^2^	250 A/cm^2^	1 kA/cm^2^
Minimum JJ size	1.2 μm×1.2 μm (with OPC)	1.2 μm×1.2 μm (with OPC)	1.2 μm×1.2 μm (with OPC)
Shrinkage of JJ size	0.2 μm	0.2 μm	0.2 μm
Device structure of JJ	RC type	RC type	RC type
Material of RES	Mo	Ti/Pd/Ti	Ti/Pd/Ti
Sheet resistance of RES	2.4 Ω	1.2 Ω	1.2 Ω
Minimum line width	1.0 μm	1.0 μm	1.0 μm
Minimum via size	0.8 μm×0.8 μm	0.8 μm×0.8 μm	0.8 μm×0.8 μm
Alignment margin	0.25 μm	0.25 μm	0.25 μm

**Table 2:** Summary of the junction characteristics measured at 4.2 K for the wafer with a lot number of 1KP001 No. 1.

TEG name	1.2-μm JJ	1.6-μm JJ	2.2-μm JJ	3.2-μm JJ	4.2-μm JJ	5.2-μm JJ	6.2-μm JJ
Junction area including shrinkage (μm^2^)	0.91	1.84	3.83	8.74	15.65	24.57	35.48
Standard deviation of *I*_c_ scattering (%)	5.17	3.08	1.69	1.19	0.74	0.53	0.53
Critical current *I*_c_ at 1.5 V (mA)	0.011	0.023	0.048	0.109	0.197	0.307	0.443
Normal current *I*_n_ at 4.0 V (mA)	0.034	0.063	0.125	0.272	0.474	0.744	1.065
*I*_c_*R*_n_ (mV)	1.344	1.455	1.525	1.598	1.660	1.651	1.663
Sub-gap resistance *R*_sg_ (Ω)	4418	2358	1187	648	329	222	146
*I*_c_*R*_sg_ (mV)	50.43	54.32	56.69	70.48	64.67	68.27	64.49
Gap voltage *V*_g_ (mV)	2.76	2.77	2.77	2.76	2.77	2.77	2.76

### RSFQ cell library using the 1KP

**Fig. 3:**
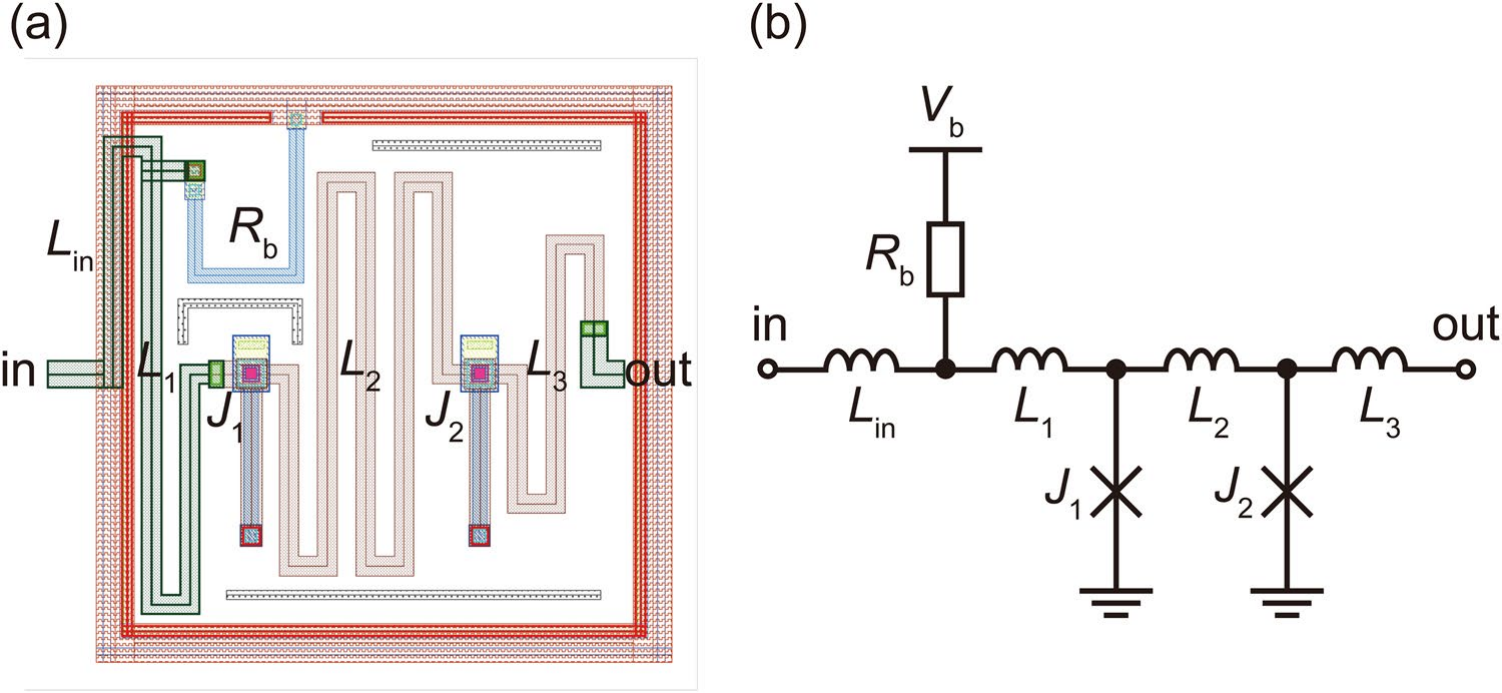
RSFQ JTL cell. (**a**) Layout. Dimensions are 60 μm (width) × 60 μm (height). (**b**) Schematic diagram. *L*_in_ = 7.36 pH, *L*_1_ = 24.3 pH, *L*_2_ = 47.8 pH, *L*_3_ = 17.3 pH, *R*_b_ = 26.7 Ω, *V*_b_ = 0.8 mV. The critical currents of *J*_1_ and *J*_2_ are 21.3 μA.

We developed an RSFQ cell library for the 1KP based on that for the PHSTP, in accordance with the design guidelines established in the CONNECT cell library^31^. As a first demonstration, we designed a minimal set of RSFQ cells: Josephson transmission lines (JTLs), a pulse splitter (SPL), an SPL with a three-fanout (SPL3), a confluence buffer (CB), a D flip-flop (DFF), a resettable DFF (RDFF), a dual-output DFF (D2FF), a non-destructive readout (NDRO), an NDRO with a complementary output (NDROC), a NOT gate, an XOR gate, a NOR gate, an OR gate, an AND gate, a T flip-flop (TFF), a resettable TFF (RTFF), a T1 flip-flop (T1FF), and DC/single-flux-quantum (SFQ) and SFQ/DC converters. Other logic cells will be designed in future work. Figures 3 (a) and (b) respectively show the layout and schematic diagram of the JTL cell. The critical currents of Josephson junctions (*J*_1_ and *J*_2_) are 21.3 µA, which is ~1/10 of those in conventional designs^7,31,34^. The circuit parameters were extracted using InductEx^36^ and are given in the caption of Fig. 3, where each inductance was designed to be ~10 times larger than that of conventional designs to keep the *LI*_c_ product constant. The McCumber parameter^37^ (*β*_c_) for each junction was set to 1. The bias voltage was reduced from a typical value of 2.5 mV to 0.8 mV to lower power dissipation, as explained later. The maximum operating frequency was estimated to be ~20 GHz^29^, which is high enough for qubit interface circuits and stochastic electronics. This maximum operating frequency is relatively conservative^29^, so RSFQ cells using the 1KP might operate at higher frequencies. The bias-feeding lines were covered with superconducting shield (SUSHI) structures^38^ to suppress magnetic fields induced by bias currents. The unit cell size was set to 60 μm × 60 μm.

We show that RSFQ cells using the 1KP can operate with very low power dissipation. The power dissipation of an RSFQ cell is given by *I*_b_*V*_b_ = 0.7*nI*_c_*V*_b_, where *I*_b_ is the bias current, *V*_b_ is the bias voltage (assuming that each junction is biased by a current equal to 70% of its *I*_c_ value), and *n* is the junction count. This is because the static power dissipation caused by bias resistors is dominant in the power dissipation of RSFQ logic. In this study, *I*_c_ values in RSFQ cells were set to 1/10 values of conventional designs. Furthermore, the *V*_b_ value was reduced to 0.8 mV, which is 32% of that for conventional RSFQ cells (2.5 mV). This is because the height of an SFQ pulse decreases with decreasing *J*_c_, so RSFQ circuits for the 1KP can operate with a low bias voltage. Consequently, the power dissipation of RSFQ cells using the 1KP is only 3.2% of that of conventional RSFQ cells. Here we clarify the impact of the 1KP on the energy efficiency of RSFQ-based qubit interface circuits and stochastic electronics. For instance, the power dissipation of a microwave pulse generator^39^ for qubit control can decrease from 51.7 µW to 1.65 µW, and that of a sigmoid function generator^40^ for superconducting neural networks can decrease from 1.7 µW to 54 nW. The power dissipation of RSFQ circuits using the 1KP can be further reduced by introducing low-power RSFQ techniques, such as energy-efficient RSFQ logic^2^, which will be investigated in future work.

### AQFP cell library using the 1KP

**Fig. 4:**
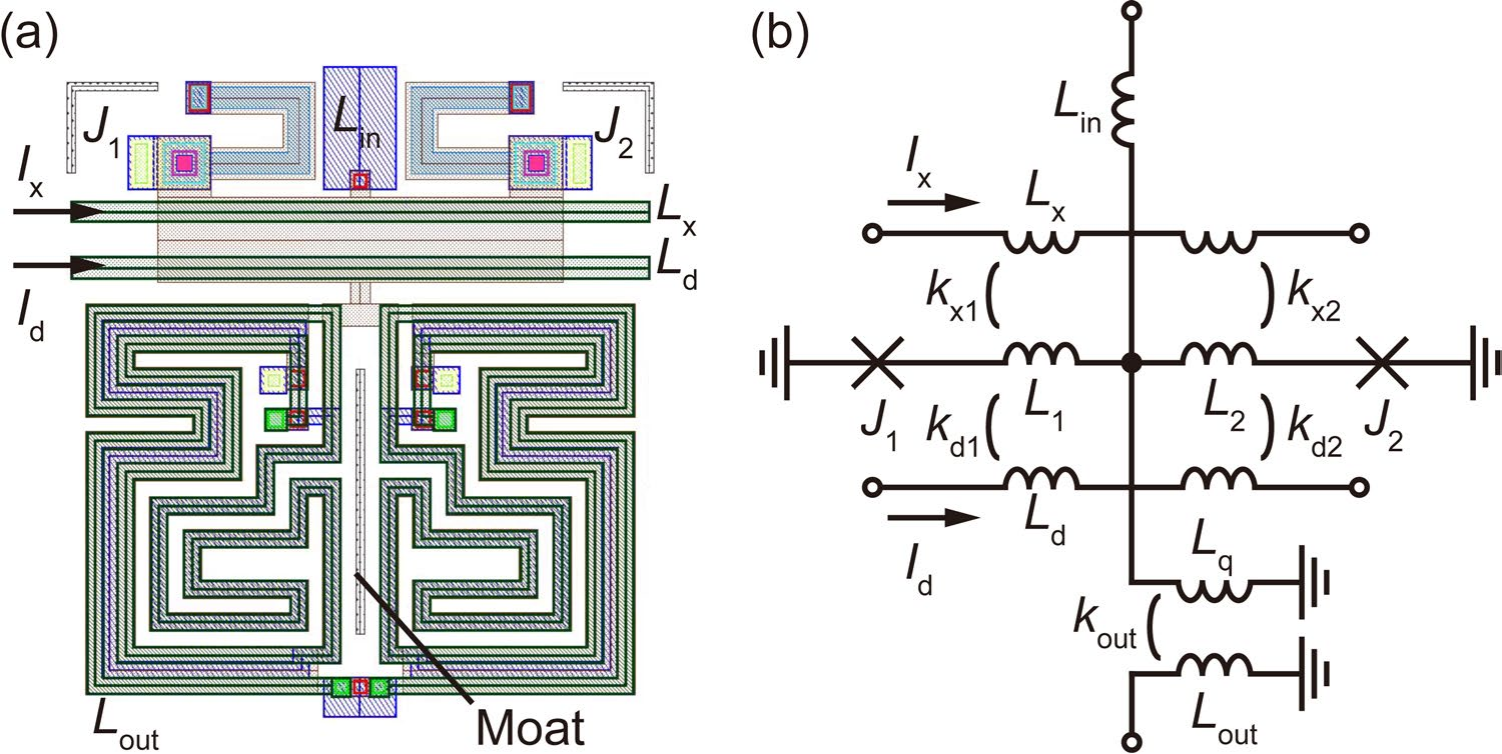
AQFP buffer cell. (**a**) Layout. Dimensions are 40 μm (width) × 45 μm (height). (**b**) Schematic diagram. *L*_in_ = 1.69 pH, *L*_1_ = *L*_2_ = 2.40 pH, *L*_q_ = 19.1 pH, *L*_out_ = 76.1 pH, *k*_out_ = 0.527. The critical currents of *J*_1_ and *J*_2_ are 20 μA.

We developed an AQFP cell library for the 1KP by adopting the minimal design^32^, in which logic gates are designed by arraying four types of building block cells (buffer, inverter, constant, and branch cells). Figures 4 (a) and (b) respectively show the layout and schematic diagram of the buffer cell, which is the most fundamental AQFP gate that transmits input data to an output port. The critical current of paired Josephson junctions (*J*_1_ and *J*_2_) is 20 µA, which is less than half of that in previous designs (50 µA)^32–34^. The other circuit parameters were extracted using InductEx and are given in the caption of Fig. 4, where the characteristic impedance of the excitation lines (*L*_x_ for applying ac excitation flux with a 0.5Φ_0_-amplitude by *I*_x_; *L*_d_ for applying DC offset flux of 0.5Φ_0_ by *I*_d_) was designed to be 50 Ω^41^. The entire layout was symmetrically designed to avoid unwanted parasitic magnetic coupling^32^ and has dimensions of 40 μm (width) and 45 μm (height). A moat^42^ for mitigating the effect of flux trapping^43,44^ was placed at the center of the output inductor *L*_out_ so that the magnetic fluxes applied by trapped fluxes to *L*_out_ would be canceled out^45^. As with the buffer cell for a 2.5 kA cm^−2^ process^46^, shunt resistors were added to *J*_1_ and *J*_2_ such that *β*_c_ for each junction became 1. This is because the sub-gap resistance of each Josephson junction is large (i.e., intrinsic damping is weak), as shown in Table 2, and shunt resistors are required to operate AQFP circuits at ~5 GHz. The maximum operating frequency was estimated to be ~30 GHz^47^, which is high enough for qubit interface circuits and stochastic electronics. The inverter and constant cells were also designed based on the buffer cell shown in Fig. 4 .

We clarify the benefits and drawbacks of AQFP circuits designed using the 1KP. A benefit is that the amplitude of excitation currents can be reduced because of large coupling between AQFP circuits and excitation lines. For the buffer cell shown in Fig.4, *J*_1_ and *J*_2_ have a small critical current of 20 µA, so the inductance of *L*_1_ and *L*_2_ was set to a larger value than previous design. This resulted in large mutual inductances between *L*_x_ and *L*_1_ (*L*_2_) and between *L*_d_ and *L*_1_ (*L*_2_), and the amplitudes of *I*_x_ and *I*_d_ were respectively reduced to 315 µA and 371 µA, which are only 40% and 33% of those for a conventional buffer cell^33^. This is important in some applications using cryocoolers, such as qubit interface circuits, because a large supply current can generate serious Joule heating through parasitic resistances and/or attenuators inside a cryocooler. A drawback of using the 1KP is that the energy dissipation of AQFP circuits is relatively large, regardless of small critical currents. The energy dissipation per switching of an AQFP buffer is given by^13^ *E*_sw_ ~ 2Φ_0_^2^/*Rτ*_x_, where *R* is the equivalent resistance of the sub-gap resistance *R*_sg_ and shunt resistor *R*_s_, and *τ*_x_ is the rise/fall time of the excitation current. This indicates that the energy dissipation is determined by the equivalent resistance, rather than critical currents. In the previous design using a 10 kA cm^−2^ process^33^, Josephson junctions were not damped by shunt resistors because of relatively strong intrinsic damping; thus, *E*_sw_ ~ 2Φ_0_^2^/*R*_sg_*τ*_x_ ~ 4 × 10^−22^J assuming *τ*_x_ = 100 ps and *R*_sg_ = 200 Ω for *I*_c_ = 50 µA. On the other hand, for the 1KP, Josephson junctions are damped by shunt resistors, so *E*_sw_ ~ 2Φ_0_^2^/*R*_s_*τ*_x_ ~ 7 × 10^−21^J assuming *τ*_x_ = 100 ps and *R*_s_ = 12.8 Ω for *I*_c_ = 20 µA (*β*_c_ = 1). The actual *E*_sw_ value of the AQFP buffer cell for the 1KP was calculated by JoSIM^48^ and found to be 8.0 × 10^−21^J for 5 GHz operation.

**Fig. 5:**
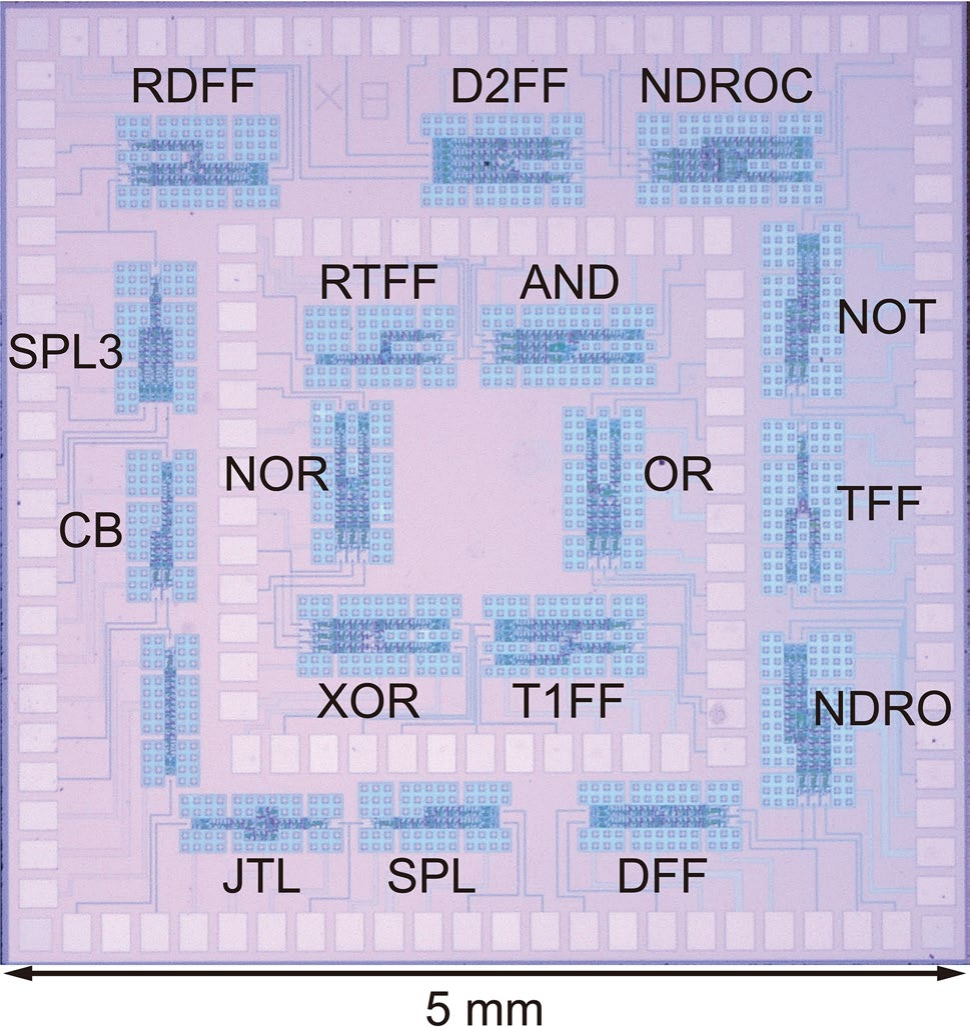
Micrograph of a test chip for the RSFQ cell library, including 17 logic cells. The chip size is 5 mm × 5 mm.

**Fig. 6:**
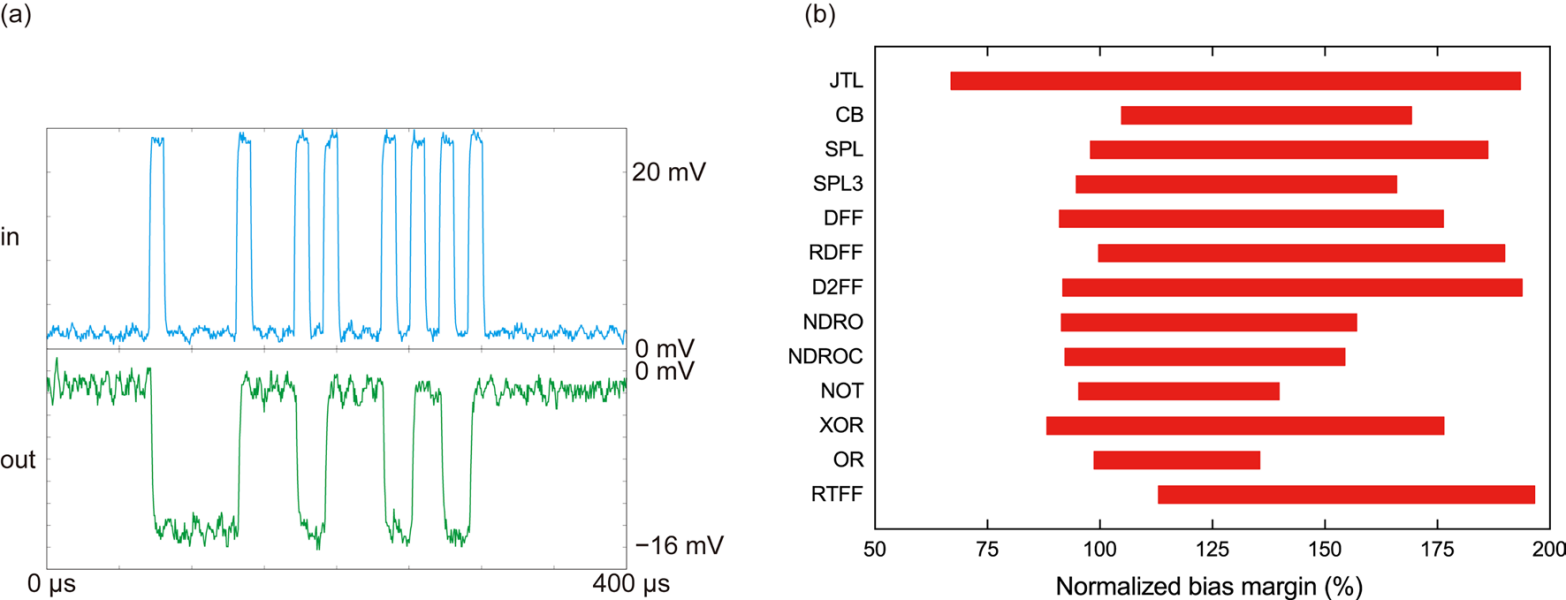
(**a**) Measurement waveforms of a JTL test circuit at 100 kHz in liquid helium. The input signal (high voltage: logic 1; low voltage: logic 0) is applied to the DC/SFQ converter, where an SFQ pulse is generated at the rise edge of the input signal. The output signal is observed by the SFQ/DC converter, where each rise or fall edge indicates a logic 1. The output signal was amplified by a room-temperature amplifier with a 40-dB gain. (**b**) Measurement results of operating bias margins, which are normalized to a bias voltage of 0.8 mV.

While the energy dissipation of the AQFP cell increased using the 1KP, the energy dissipation is still very small compared to that of typical superconductor logic families (10^−19^J to 10^−17^J)^1,2,4^; also, the energy dissipation can be further reduced by increasing *β*_c_ values. More importantly, with the low *J*_c_ value, a wide range of *I*_c_ values (10–50 µA) are available to balance the power dissipation and amount of supply currents for cryocooler applications. Another important point is that the power dissipation of some AQFP/RSFQ hybrid systems, such as a qubit calibration circuit^16^ and neuron circuit^40^, can be significantly reduced using the 1KP because the entire power dissipation of hybrid systems is typically dominated by the RSFQ parts.

### Measurement results

We fabricated RSFQ and AQFP logic cells using the 1KP and demonstrated the cells at 4.2 K in liquid helium using a low-frequency dipping probe with multiple magnetic shields. The operating frequencies of microwave pulse generators for qubit interface circuits are ~5 GHz^18,39^, so we will test RSFQ and AQFP circuits using the 1KP at frequencies in the gigahertz range in future work.

**Fig. 7:**
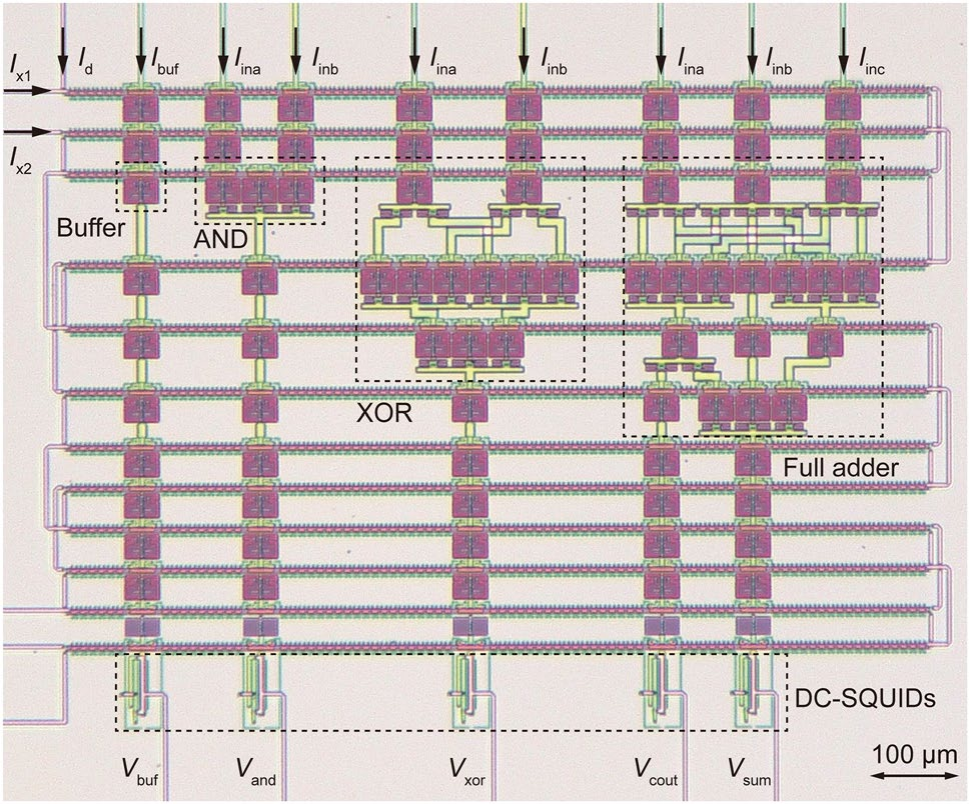
Micrograph of AQFP circuits that include a buffer chain, an AND gate, an XOR gate, and a full adder. The entire circuit was powered and clocked by paired ac excitation currents (*I*_x1_ and *I*_x2_) and a DC offset current (*I*_d_).

**Fig. 8:**
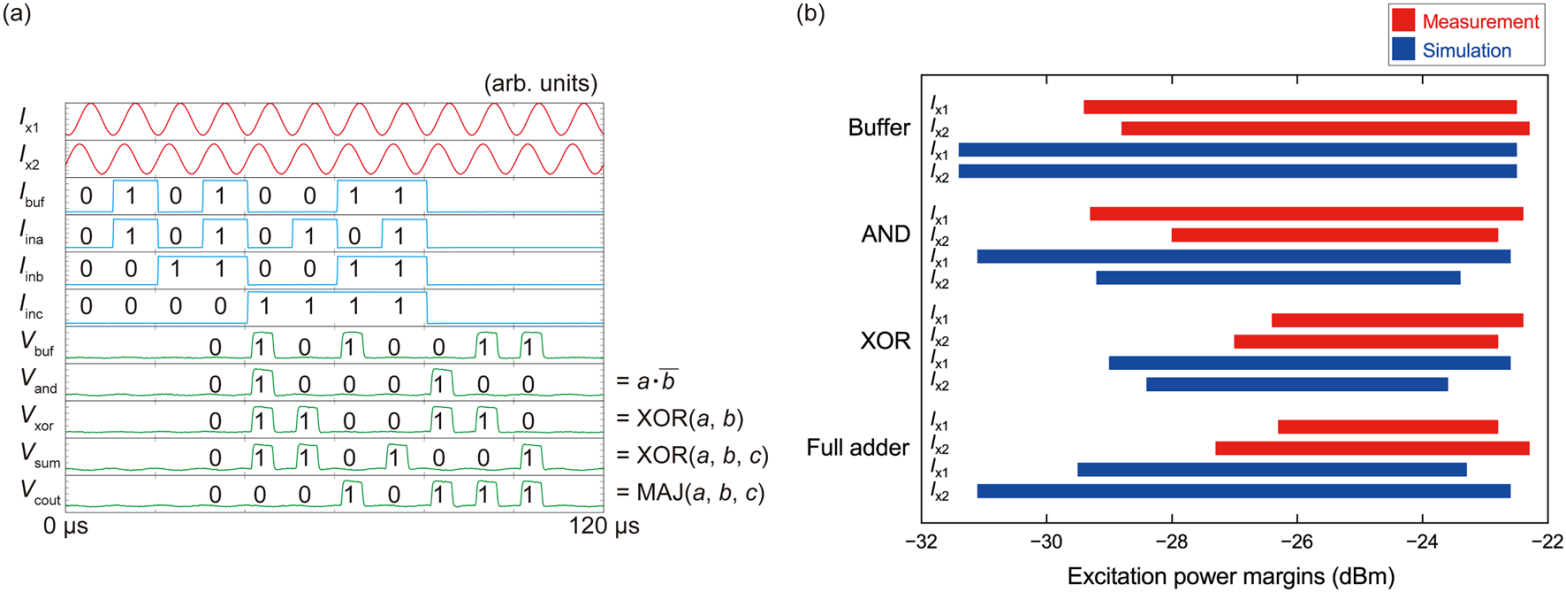
(**a**) Measurement waveforms obtained at 100 kHz in liquid helium. All the circuits operated correctly. (**b**) Measurement and simulation results of operating margins, where thermal noise was not taken into account in the simulation.

### Demonstration of RSFQ cells

Figure 5 shows a micrograph of a test chip for the RSFQ cell library fabricated by the 1KP, which includes the above-mentioned 17 logic cells. DC/SFQ and SFQ/DC converters were used as input and output interfaces between each logic cell and room temperature electronics. We tested the logic cells at a low frequency (100 kHz) and found that 13 cells operated correctly. Figure 6 (a) shows measurement waveforms of the JTL, and Fig. 6 (b) shows measurement results of operating margins regarding the bias voltage, normalized by the design value (0.8 mV). The bias margins shifted to a higher region because *J*_c_ of the fabricated chip was larger than the design value by ~20%. All the cells except for NOT and OR gates operated with wide bias margins of more than 60%, but the NOT and OR gates still exhibited reasonably wide bias margins of more than 35%. Unfortunately, the AND, NOR, TFF, and T1FF cells did not operate correctly. As for the AND gate, there were some mistakes in the parameter design and layout. For the other unfunctional cells, error rates were too high to evaluate bias margins due to thermal noise. This indicates that some cells require design improvements.

### Demonstration of AQFP cells

Figure 7 shows a micrograph of AQFP circuits fabricated by the 1KP. The circuits include a buffer chain, an AND gate, an XOR gate, and a full adder. The circuits were powered and clocked by a pair of sinusoidal excitation currents (*I*_x1_ and *I*_x2_) with a phase separation of 90° and a DC offset current *I*_d_. In this way, logic operations were performed with a phase separation of 90°^33,49^. *I*_buf_ is the input current for the buffer chain; *I*_ina_ and *I*_inb_ are the input currents for the AND gate, XOR gate, and full adder; and *I*_inc_ is the input current for the full adder. The amplitude of *I*_buf_, *I*_ina_, *I*_inb_, and *I*_inc_ was 10 µA. *V*_buf_, *V*_and_, and *V*_xor_ are the output voltages for the buffer chain, AND gate, and XOR gate, respectively; and *V*_cout_ and *V*_sum_ are the output voltages representing the carry-out and summation for the full adder, respectively. The output voltages were generated by DC superconducting quantum interference devices (SQUIDs) coupled to AQFP buffers^5^. Figure 8 (a) shows measurement waveforms of the AQFP circuits at 100 kHz, and Fig. 8 (b) compares measured and simulated operating margins for *I*_x1_ and *I*_x2_, where thermal noise was not taken into account in the simulation. Compared to the simulated margins, the measured margins may have decreased because the critical current of Josephson junctions (20 µA) was small for error-free operation at 4.2 K; however, this is not problematic for our purpose since qubit interface circuits operate at much lower temperatures, and stochastic electronics accept errors to some extent.

## Conclusions

We developed the 1KP for the design of qubit interface circuits and stochastic electronics, and developed RSFQ and AQFP cell libraries using the 1KP. The power dissipation of RSFQ logic using the 1KP was reduced to 3.2% of that for conventional RSFQ logic by reducing the *I*_c_ values of Josephson junctions and bias voltage. Also, the amount of supply currents for AQFP circuits was reduced to ~40% of that for conventional AQFP circuits due to large mutual inductances between AQFP gates and excitation lines. These two points are beneficial on the development of qubit interface circuits and stochastic electronics. We demonstrated both RSFQ and AQFP circuits fabricated by the 1KP at 4.2 K. Our next step is to test the circuits at even lower temperatures such as 10 mK to clearly show that RSFQ and AQFP logic using the 1KP can be used for qubit interface circuits and stochastic electronics.

## Data Availability

The data that support the findings of this study are available from the corresponding authors upon reasonable request.
